# Case reports of cerebral sinus venous thrombosis in COVID-19 patients

**DOI:** 10.1186/s41983-021-00335-y

**Published:** 2021-06-29

**Authors:** Prysta Aderlia Sitanggang, Kumara Tini, Ni Made Susilawathi, Ida Ayu Sri Wijayanti, Putu Utami Dewi, Dewa Putu Gde Purwa Samatra

**Affiliations:** 1 Departement of Neurology, Udayana University, Sanglah General Hospital, Jalan Kesehatan No.1, Denpasar, Bali Indonesia; 2 Department of Neurology, Faculty of Medicine, Universitas Udayana, Universitas Udayana Hospital, Jalan Rumah Sakit Universitas Udayana No.1, Jimbaran, South Kuta, Bali Badung, Indonesia; 3 Department of Radiology, Faculty of Medicine, Universitas Udayana, Universitas Udayana Hospital, Jalan Rumah Sakit Universitas Udayana No.1, Jimbaran, South Kuta, Badung, Bali Indonesia

**Keywords:** COVID 19, CSVT, Thrombogenesis, Coagulation cascade

## Abstract

**Background:**

Coronavirus disease 2019 (COVID-19) pandemic has started in December 2019 and still ongoing. The disease has been expanding rapidly with a high variety of phenotypes from asymptomatic, mild respiratory tract infection, multiple organ system dysfunction, and death. Neurological manifestations also appear in patients with COVID-19, such as headache, seizures, a decrease of consciousness, and paralysis. The hypercoagulable state in patients with COVID-19 is associated with the thromboembolic incident including ischemic strokes, venous thromboembolism, pulmonary artery embolism, and many further. Cerebral sinus venous thrombosis (CSVT) is a rare neurovascular emergency that is often found in critically ill patients. We report two cases of CSVT with different onsets, neurologic manifestations, and prognoses.

**Case presentation:**

Two cases of cerebral sinus venous thrombosis in COVID-19 patients were reported, following respiratory, hematology, and coagulation disarrangements, which was triggered by the severe acute respiratory syndrome coronavirus 2 (SARS-CoV-2) infection. The first patient, which was presented with a seizure, had hypertension and diabetes mellitus as comorbidities. The latter case had no comorbidity but showed more severe presentations of COVID-19 such as brain and lung thrombosis, although already had several days of intravenous anticoagulant administrations. These two cases also have a different course of disease and outcomes, which were interesting topics to study.

**Conclusions:**

CSVT is one of the neurological complications of the COVID-19 when the brainstem venous drainage is involved. Despite successful alteration to the negative result of SARS-CoV-2 through the rt-PCR test, thrombogenesis and coagulation cascade continuing. Therefore, a high level of neutrophil to lymphocyte ratio (NLR), D-dimer, fibrinogen, and C-reactive protein (CRP) are paramount indicators of poor prognosis.

## Background

In December 2019, the cases of SARS-CoV-2 infection have been observed to cause severe pneumonia, in Hubei Province, China [1]. Several studies have described the clinical manifestation in COVID-19 including fever, cough, malaise, dyspnea, and decrease of consciousness [2–4]. COVID-19 involvement in the nervous system was also reported with cerebrovascular disease, headache, impaired consciousness, seizure, impairment of smell, and skeletal muscle injury [5]. Critically ill patients are prone to have neurological manifestations with some comorbidities of older age, hypertension, diabetes, and/or cardiac disease [5]. Ischemic and hemorrhagic stroke also reported as cerebrovascular disease (CVD) in severe COVID-19 [6]. The CVS in COVID-19 may be due to high levels of hypercoagulable state after the cytokine storm. Massive thrombosis in COVID-19 patients may occlude the venous system in the brain that ended with cerebral sinus venous thrombosis (CSVT) [7–9]. The CSVT is a rare neurovascular emergency but further investigation in these cases may help to prevent the delay in diagnosing and treating these cases in the COVID-19 pandemic.

We reported two cases of concomitant CSVT and COVID-19 infection. The patients developed a profound neurological injury, secondary to CSVT, with SARS-CoV-2 infection confirmed by the reverse transcriptase-polymerase chain reaction (rt-PCR) test. The SARS-CoV-2 virus activates the inflammatory cascade and thrombotic pathways, by binding angiotensin-converting enzyme 2 (ACE2) receptors of endothelial cells [10]. This leads to a hypercoagulable state, proved by the increase of D-dimer [3, 11]. Furthermore, the increased level of D-dimer indicates high thrombus formation [12], although two cases of CSVT were having a neurological manifestation of COVID-19.

## Case presentation

On the 5th of August, 2020, a 58-year-old woman presented in our emergency department with seizures and hemiparesis. She had a prior fever, malaise, and dry cough a week before admission. She has had a history of hypertension since a year ago. The vital sign is within normal limit except the blood pressure is 170/110 mmHg.

The results of the laboratory test were high blood glucose 254 mg/dL and HbA1C 6.5%. Leukocytosis with white blood cells (WBC) was 14/L and neutrophil-lymphocyte ratio (NLR) was 33. Increase level of erythrocyte sedimentation rate (ESR) 93 mm/h, D-dimer 21,176 ng/mL, and fibrinogen 446 mg/dL. The result of the reverse transcription-polymerase chain reaction (rt-PCR) test from the nasal swab was observed to be positive for SARS-CoV-2. The completed laboratory results are listed in Table 1

**Table 1:** Summary of laboratory findings at admission

Laboratory findings	Case 1	Case 2
**White cell count (per mm**^**3**^**)**	**14.1**	**53.03**
**Total neutrophils (per mm**^**3**^**)**	**13.02**	**49.53**
**Total lymphocytes (per mm**^**3**^**)**	**0.39**	**0.84**
**Neutrophil-Lymphocyte Ratio**	**33**	**59**
**Total monocytes (per mm**^**3**^**)**	**0.58**	**1.35**
**Hemoglobin level (g/dL)**	**14.1**	**15.0**
**Hematocrit**	**40.4**	**44.3**
**Platelet count (per mm**^**3**^**)**	**311**	**397**
**ESR**	**93**	**-**
**C-reactive protein (mg/L)**	**-**	**221.1 (before 391)**
**Procalcitonin (mg/L)**	**-**	**7.76**
**Lactate dehydrogenase (U/L)**	**-**	**600**
**Activated partial thromboplastin time (s)**	**27.4**	**20.2**
**Prothrombin time (s)**	**9.3**	**11.9**
**INR**	**0.90**	**1.11**
**D-dimer level (ng/mL)**	**21,176**	**5,308 (before > 50.000)**
**Fibrinogen level (mg/dL)**	**446**	**525 (before 439)**
**Albumin level (g/dL)**	**2.71**	**-**
**SGOT (U/L)**	**29**	**169**
**SGPT (U/L)**	**24**	**238**
**Blood urea nitrogen (mg/dL)**	**14**	**31**
**Serum creatinine (mg/dL)**	**0.74**	**0.52**
**Blood glucose**	**254**	**130**
**HbA1C**	**6.5**	**-**
**Natrium (mg/dL)**	**133**	**139**
**Kalium (mg/dL)**	**3.0**	**4.5**
**Calcium (mg/dL)**	**7.4**	**7.6**
**Magnesium (mg/dL)**	**2.17**	**2.2**
**CKMB**	**-**	**31.8**
**Troponin I**	**-**	**0.61**
**Blood gas analysis:**		
- **pH**	**7.42**	**7.29**
- **pCO**_**2**_ **(mmHg)**	**40**	**45**
- **pO**_**2**_ **(mmHg)**	**136**	**92**
- **Base excess (mmol)**	**2**	**− 4**
- **Lactate (mmol/L)**	**-**	**1.8**

*ESR* Erythrocyte sedimentation rate, *INR* International normalized ratio, *SGOT* Serum glutamic oxaloacetic transaminase, *SGPT* Serum glutamic pyruvic transaminase, *HbA1c* Hemoglobin A1c, *CKMB* Creatinine kinase myocardial band

**Fig. 1:**
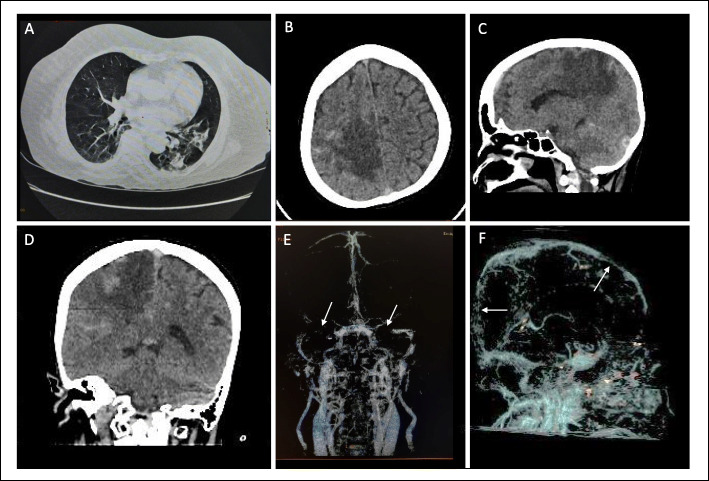
Acute onset of cerebral sinus venous thrombosis in COVID-19. **A** Axial thorax CT-scan showed ground-glass opacity and subpleural bilateral, with a fibrotic appearance in the left lung. **B** Axial head non-contrast CT-scan (NCCT scan) showed, parenchymal edema on the right parietal lobe and hyperdense cortical veins. **C** Sagittal NCCT scan showed parenchymal edema on the right parietal lobe and hyperdense superior sagittal sinus. **D** Coronal head NCCT scan showed parenchymal edema on the right parietal lobe and hyperdense superior sagittal sinus. **E**, **F** Brain CT venography showed, filling defect at SSS, TS bilateral, sigmoid sinus bilateral

Head contrast computed tomography (HCCT) showed cerebral edema at the right parietal lobe (Fig. 1B). There were hyperdense veins and sinuses at the right parietal lobe area, superior sagittal sinus (SSS), and bilateral transverse sinuses (TS), which suggested the presence of a cerebral sinus venous thrombosis (CSVT) (Fig. 1B–D), with a sign of venal hypertension. CT venography (CTV) was also confirmed CSVT at SSS, bilateral TS, and bilateral sigmoid sinus (Fig. 1E, F). Furthermore, the thorax CT scan showed ground-glass opacity and subpleural bilateral, with a fibrotic appearance in the left lung (Fig. 1A).

During the patient’s treatment of COVID-19, 60 mg subcutaneous enoxaparin was administered twice daily, for 5 days. On the 5th day of treatment, D-dimer was decreased to 2092 ng/ml. The decision was made to continue with enoxaparin until the normal D-dimer or the patient’s negative status for the COVID-19 virus is attained. Other clinical manifestations also improved and the patient was discharged from the hospital on the 10th day.

The second case, a 72-year-old male, was consulted from the intensive care unit (ICU) because of sudden decreases in consciousness. He had a history of high fever, dry cough, and shortness of breath a week before hospitalization. The patient was being treated as severe COVID-19 with ARDS for 10 days in ICU. He had no previous history of hypertension, diabetes mellitus, or cardiovascular disease. He already received heparin intravenously for 10 days and halted, due to good recovery of shortness of breath and hypoxemia. A day after heparin cessation, the patient suddenly unconscious and intubated due to severe hypoxia.

Upon examination, his consciousness is coma with a slow response to the light of both eyes. He had hypothermia with a temperature of 35.4 °C, blood pressure of 140/69 mmHg on vasoconstrictor epinephrine support, and 90% oxygen saturation.

Laboratory test showed, critical increase level of WBC (53.09 × 10^3^/L) and NLR 58.1. We did not do a peripheral blood smear to examine any hematological disorder. Coagulation function tests were APTT 20.2 s, PT 11.9 s, INR 1.11, D-dimer 5.308 ng/mL (previous was > 50,000), and fibrinogen 525 mg/dL. The rt-PCR test from a nasopharyngeal swab sample was negative for SARS-CoV-2 on the 11th day. The laboratory results are listed in Table 1.

**Fig. 2:**
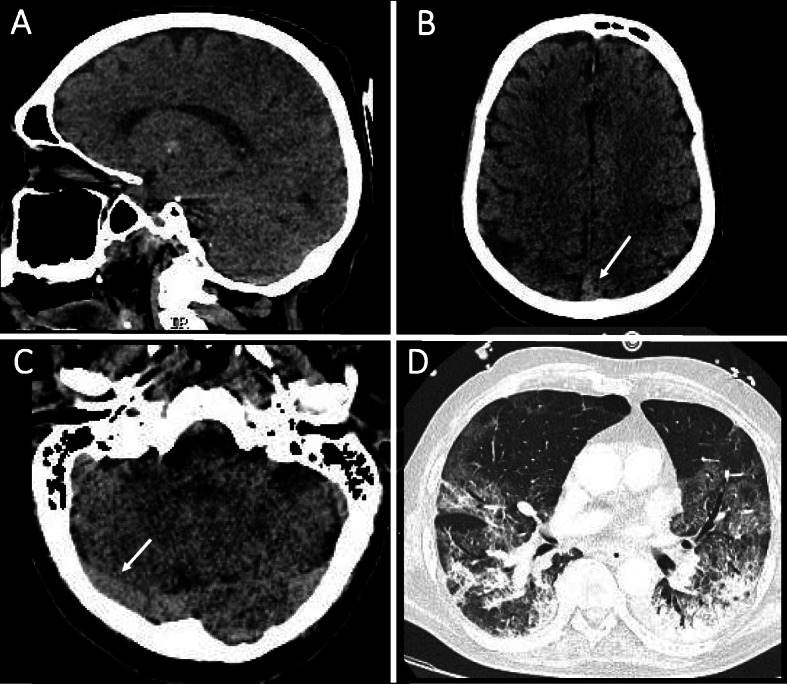
Subacute onset of cerebral sinus venous thrombosis in a man with COVID-19 and ARDS. **A** Sagittal head NCCT scan showed hyperdense at superior sagittal sinus and right transverse sinuses. **B** Axial head NCCT scan showed hyperdense at superior sagittal sinus (white arrow) and brain parenchymal edema on the left parietal lobe. **C** Axial head NCCT scan showed hyperdense at right transverse sinus (white arrow). **D** Axial thorax CT-scan showed ground-glass opacity and subpleural bilateral, with the fibrotic appearance in both lungs

Head non-contrast computed tomography (NCCT) showed hyperdense at superior sagittal sinus and right transverse sinuses, which suggested the manifestations of CSVT (Fig. 2A–C). Furthermore, there was associated cerebral edema at the left frontal, parietal lobe, and cerebellum (Fig. 2B). Thorax CT scan showed ground-glass opacity and subpleural bilateral with the fibrotic appearance in both lung and pulmonary angiography. Thrombosis at segments 8 and 9 of both lungs was also observed (Fig. 2D). The pulmonary CT-angiography (CTA) results were pulmonary embolism and thrombosis of both the left and right pulmonary arteries. Due to the massive thrombosis, a decision was made to administer streptokinase and heparin, to the patient intravenously. After 3 days of this treatment, the patient became desaturated (40% oxygen saturation) with spontaneous pneumothorax and passed away the next day.

## Discussion

CSVT is a rare cerebrovascular disease that incidences only 0.5% to 1% of all stroke cases [13–15]. CSVT is more common in young individuals, with broad manifestation, depending on the site of the thrombosis [13, 16]. It is concomitant with multiple factors, for example prior medical conditions (thrombophilia, inflammatory bowel disease), transient situations (pregnancy, dehydration, infection), particular medications (oral contraceptives, substance abuse), and unpredictable events (head trauma) [15], with which thrombus formation becomes more intensified. The poor outcomes and prognosis of CSVT are associated with, age, gender, and medical conditions [15].

Cases from our reports have different neurological manifestations after the previous episode of upper respiratory tract symptoms with confirmed SARS-CoV-2 infection from nasopharyngeal swab rt-PCR. SARS-CoV-2 is transmitted mostly through respiratory droplets and infected the lungs [17]. The entry of SARS-CoV-2 into the human host cells relies on the surface angiotensin-converting enzyme 2 (ACE2), which is most expressed in the type II surfactant-secreting alveolar cells of the lungs [18]. The incubation period is approximately 4–5 days before the onset of symptoms [19, 20]. Almost all patients with COVID-19 have lung problems, such as cough, shortness of breath, or ARDS [19]. Guan and colleagues reported that the clinical outcomes of COVID-19 can be predicted by the amount of comorbidities for example hypertension, diabetes mellitus, cardiovascular disease, stroke, smoking, and age more than years old [21]. The first patient had two comorbidities of hypertension and diabetes mellitus, while the second patient has age > 60 years old as comorbid.

SARS-CoV-2 infection activates disproportionate inflammatory innate and adaptive immune response [22]. The interleukin (IL)-1β, IL-6, IL-12, interferon γ (IFN-γ), IFN-γ-inducible protein 10 (IP10), and monocyte chemoattractant protein (MCP) were associated with pulmonary inflammation and extensive lung damage in SARS-CoV-2 patients [23]. Both patients had severe pneumonia with GGO in their thorax CT scan. The worse clinical experience in the second patient was most likely the result of this immunity instability. Furthermore, elderly patients are more susceptible to being worsened against viral infections, due to decreased interferon production [24]. SARS-CoV-2 is known to suppress the induction of antiviral type I interferon (IFN-α/β) [24].

“Cytokine storm” has been a major cause of severe COVID-19 with high levels of inflammatory markers found in the blood (C-reactive protein, ferritin, and D-dimers) [25, 26]. Hematologic examination in severe COVID-19 showed lymphocytopenia and an increased sum of neutrophils (NEU) [27]. Neutrophils are activated and migrated to the infected. Elevation of the NLR predicts the adverse prognostic in COVID-19 patients [28]. Both patients had NLR of more than 5, especially the second patient had twofold NLR than the first patient. Extremely high leukocyte level is a bad predictor in the second patient. NEU induces DNA cell damage by releasing reactive oxygen species [28]. A study published by Huang and colleagues reported leukocytosis 2.0 rise and neutrophilia 4.4-fold rise [29] as predictor severity of the disease. This may be confused in myeloproliferative neoplasm (MPN), but we did not do the blood film examination to exclude the disease in this patient. The pathogenesis of thrombosis in MPN is related to an increase in blood cell counts (leukocytosis, erythrocytosis, and thrombocytosis) and the presence of JAK2 mutation. Patients with WBC > 15 × 10^9^/L was a significant predictor for thrombosis [30].

Clinically, relevant hemostatic changes occur 50–70% in septic patients, with 35% meeting the criteria of disseminated intravascular coagulation [31]. Furthermore, patients with COVID-19 have diffused inflammation, which activates the coagulation system by consuming clotting factors and resulting in DIC [32]. The systemic infection of SARS-CoV-2 damages the endothelial cells, activates mononuclear cells, produces proinflammatory cytokines, and promotes coagulation [33]. Thrombin elicits the production of chemoattractant proteins, in monocytes, fibroblasts, mesothelial, and vascular endothelial cells, by interacting with protease-activated receptors (PARs) 1,3, and 4 [31]. PARs are transmembrane G-protein coupled receptors that have their functions from 1–4 [34]. The PARs 1, 3, and 4 are receptors activated by thrombin. Through PAR2, factor Xa and the tissue factor (VIIa) complex also upregulate chemoattractant proteins (IL-6, IL-8) in vascular endothelial cells [31]. The tissue factor (VIIa) catalyzes the conversion of factor X to Xa, forming prothrombinase complex with factor Va, prothrombin factor (II), and calcium, which results in generating thrombin factor (IIa) [32]. The physiological anticoagulant mechanisms and fibrinolysis inhibited by endothelial cells cause intravascular fibrin deposition [31].

The mortality rate in CSVT is 1% at discharge and continuing to decrease with the use of anticoagulant treatment [14]. The main cause of early death after acute CSVT is secondary trans-tentorial herniation, due to multiple lesions to diffuse brain edema [15]. Other causes are status epilepticus, medical complications, and pulmonary embolism [15]. According to the laboratory and thorax CTA results, the second patient had pulmonary embolism and thrombosis at the same time (Table 1).

The difference of the clinical presentation’s severity in both patients was also determined by the location of vein thrombosis and the brain edema. The first patient presented better clinical manifestation, because the edema was in the supratentorial brain area, while the second exhibited worse symptoms due to involvement of infratentorial brain edema (cerebellum and pons). Head NCCT scan of the second case showed direct signs of dense triangle sign (Fig. 2B white arrow) and cord at right TS (Fig. 2C white arrow). Furthermore, the CTV of the first patient showed a filling defect at bilateral TS and SSS (Fig. 1E, F white arrows). During the COVID-19 pandemic, CTV was preferred over MRI because it is an accurate and more rapid technique to detect cerebral venous thrombosis [35]. The massive thrombosis and emboli in the lungs were the consequences of the hypercoagulable state and body immobility. Thrombosis makes up 31% of thrombotic complications in ICU patients down with COVID-19 [36]. The second patient had late thrombosis complications although had been administered by heparin, which might be related to this immobilized condition and high level of D-dimer in the first presentation.

Critically ill patients develop a hypercoagulable state due to immobilization, mechanical ventilation, central venous access devices, and nutritional deficiencies [37]. COVID-19 and hypercoagulability are further implicating the risks of pulmonary embolism (PE), vein thromboembolism (VTE), disseminated intravascular coagulation (DIC), and stroke [38]. The dysfunction of endothelial cells plays role in thrombin generation and fibrinolysis shutdown [34]. The second patient had a more severe condition, because of being hospitalized with a ventilator, central venous catheter, and immobilized for 10 days.

Pro-coagulation state in COVID-19 generates CSVT. Hypercoagulability of SARS-CoV-2 serves as an increase in, D-dimer, LDH, fibrinogen, factor VIII (FVIII), von Willebrand factor (vWF), and decreased antithrombin [39]. Patients with severe pneumonia, especially ARDS, have low oxygen concentration that increases blood viscosity, while also inducing the hypoxia-inducible transcription factor-dependent signaling pathway [36]. After the COVID-19 swab had been negative, the second patient was observed to develop massive thrombosis. The second patient had more D-dimer levels than the first, and the length of treatment was longer. Furthermore, the thrombosis process was still ongoing even though COVID-19 was negative and the D-dimer level was decreased. It was a challenge to observe whether the second patient had the previous thrombosis or not, with a diagnostic approach taken in checking for D-dimer and heart thrombosis [40]. Therefore, it was concluded that this patient had brain, heart, and pulmonary thrombosis.

Three important physiological anticoagulant pathways are the antithrombin, the activated protein C, and tissue factor inhibitor (TFPI) system. Those pathways are deranging in sepsis, which is precipitated by cytokines [34]. In sepsis, a high level of cytokines is found in the circulation, with hemostatic activation mediated by TNF. The expression of tissue factor in mononuclear cells and subsequent exposure to blood results in thrombin generation, accompanied by fibrinogen to fibrin conversion. The interaction and activation of platelets and their walls contribute to microvascular clot formation [34].

The anticoagulant commonly used in preventing DIC and VTE is low molecular weight heparin (LMWH) because it has an anti-inflammatory effect [41]. The first patient was given LMWH, to minimalize the contact with the patient. The D-dimer was diagnosed after 4 days and observed to be decreased, with improvement in neurological manifestation. Due to being in ICU, heparin was intravenously administered to the second patient, to pay close attention to the APTT. Heparin interacts with pro-inflammatory and procoagulant cascades, to prevent inflammation, and coagulopathy associated with sepsis [41]. Unfractionated heparin and LMWH are mostly used, in the cases of acute CSVT [9]. Tissue-plasminogen activator (tPA), in fibrinolytic therapy, is used in patients with low compensating conditions and resilient from prior anticoagulant [42]. The second patient was given fibrinolytic therapy the following day, after the diagnosis of CSVT. Due to multiple organ failures and loss of consciousness, it was therefore concluded that the response was poor.

## Conclusion

CSVT is one of the neurological complications of COVID-19 manifestation, possessing a grave prognosis when the brainstem venous drainage is involved. Hypercoagulability is also a complication of this disease, in patients with previous comorbid hypertension and diabetes mellitus. Thrombogenesis and coagulation cascades are prolonged, despite successful alteration to the negative result of SARS-CoV-2, through the use of the rt-PCR test. When monitoring neutrophil and lymphocyte ratio (NLR), it was observed that D-dimer level, fibrinogen, and CRP are bad prognosis indicators. Therefore, the use of heparin or LMWH decreases the D-dimer and not by any means increase the symptoms.

## Data Availability

Data sharing is not applicable to this article as no datasets were generated or analyzed during the current study.

## Abbreviations

ACE2: Angiotensin-converting enzyme 2
APTT: Activated partial thromboplastin time
ARDS: Acute respiratory distress syndrome
BUN: Blood urea nitrogen
CKMB: Creatinine kinase myocardial band
COVID-19: Coronavirus disease 2019
CRP: C-reactive protein
CSVT: Cerebral sinus venous thrombosis
CT: Computed tomography
CTA: CT angiography
CTV: CT venography
DIC: Disseminated intravascular coagulation
ESR: Erythrocyte sedimentation rate
FVIII: Factor VIII
GGO: Ground glass opacity
HbA1C: Hemoglobin A1c
HCCT: Head contrast computed tomography
ICU: Intensive care unit
IFN: Interferon
IL: Interleukin
IP10: Inducible protein 10
INR: Index normalized ratio
LDH: Lactate dehydrogenase
LMWH: Low molecular weight heparin
MCP: Monocyte chemoattractant protein
NEU: Neutrophils
NLR: Neutrophil to lymphocyte
PAR: Protease-activated receptors
PE: Pulmonary embolism
PT: Prothrombin time
SARS-CoV-2: Severe acute respiratory syndrome coronavirus 2
SGOT: Serum glutamic oxaloacetic transaminase
SGPT: Serum glutamic pyruvic transaminase
SSS: Superior sagittal sinus
TFPI: Tissue factor inhibitor
t-PA: Tissue-plasminogen activator
TS: Transverse sinus
VTE: Vein thromboembolism
vWF: von Willebrand factor
WBC: White blood cells

## References

[1] Zhu N, Zhang D, Wang W, Li X, Yang B, Song J, Zhao X, Huang B, Shi W, Lu R, Niu P, Zhan F, Ma X, Wang D, Xu W, Wu G, Gao GF, Tan W, China Novel Coronavirus Investigating and Research Team (2020). A novel coronavirus from patients with pneumonia in China, 2019. N Engl J Med..

[2] Chen N, Zhou M, Dong X, Qu J, Gong F, Han Y, Qiu Y, Wang J, Liu Y, Wei Y, Xia J', Yu T, Zhang X, Zhang L (2020). Epidemiological and clinical characteristics of 99 cases of 2019 novel coronavirus pneumonia in Wuhan, China: a descriptive study. Lancet..

[3] Wang D, Hu B, Hu C, Zhu F, Liu X, Zhang J, Wang B, Xiang H, Cheng Z, Xiong Y, Zhao Y, Li Y, Wang X, Peng Z (2020). Clinical characteristics of 138 hospitalized patients with 2019 novel coronavirus-infected pneumonia in Wuhan, China. JAMA.

[4] Liu K, Fang YY, Deng Y, Liu W, Wang MF, Ma JP, Xiao W, Wang YN, Zhong MH, Li CH, Li GC, Liu HG (2020). Clinical characteristics of novel coronavirus cases in tertiary hospitals in Hubei Province. Chin Med J (Engl)..

[5] Mao L, Jin H, Wang M, Hu Y, Chen S, He Q, Chang J, Hong C, Zhou Y, Wang D, Miao X, Li Y, Hu B (2020). Neurologic manifestations of hospitalized patients with coronavirus disease 2019 in Wuhan, China. JAMA Neurol.

[6] Katz JM, Libman RB, Wang JJ, Sanelli P, Filippi CG, Gribko M, Pacia SV, Kuzniecky RI, Najjar S, Azhar S (2020). Cerebrovascular complications of COVID-19. Stroke..

[7] Cavalcanti DD, Raz E, Shapiro M, Dehkharghani S, Yaghi S, Lillemoe K, Nossek E, Torres J, Jain R, Riina HA, Radmanesh A, Nelson PK (2020). Cerebral venous thrombosis associated with COVID-19. AJNR Am J Neuroradiol..

[8] Garaci F, Di Giuliano F, Picchi E, Da Ros V, Floris R (2020). Venous cerebral thrombosis in COVID-19 patient. J Neurol Sci..

[9] Klein DE, Libman R, Kirsch C, Arora R (2020). Cerebral venous thrombosis: a typical presentation of COVID-19 in the young. J Stroke Cerebrovasc Dis..

[10] Hamming I, Timens W, Bulthuis ML, Lely AT, Navis G, van Goor H (2004). Tissue distribution of ACE2 protein, the functional receptor for SARS coronavirus. A first step in understanding SARS pathogenesis. J Pathol..

[11] Zhou F, Yu T, Du R, Fan G, Liu Y, Liu Z (2020). Clinical course and risk factors for mortality of adult inpatients with COVID-19 in Wuhan, China: a retrospective cohort study. Lancet..

[12] Spiezia L, Boscolo A, Poletto F, Cerruti L, Tiberio I, Campello E, Navalesi P, Simioni P (2020). COVID-19-related severe hypercoagulability in patients admitted to intensive care unit for acute respiratory failure. Thromb Haemost..

[13] Devasagayam S, Wyatt B, Leyden J, Kleinig T (2016). Cerebral venous sinus thrombosis incidence is higher than previously thought: a retrospective population-based study. Stroke..

[14] Coutinho JM, Zuurbier SM, Aramideh M, Stam J (2012). The incidence of cerebral venous thrombosis: a cross-sectional study. Stroke..

[15] Saposnik G, Barinagarrementeria F, Brown RD, Bushnell CD, Cucchiara B, Cushman M (2011). Diagnosis and management of cerebral venous thrombosis: a statement for healthcare professionals from the American Heart Association/American Stroke Association. Stroke..

[16] Dmytriw AA, Song JSA, Yu E, Poon CS (2018). Cerebral venous thrombosis: state of the art diagnosis and management. Neuroradiology..

[17] Hui KPY, Cheung MC, Perera RAPM, Ng KC, Bui CHT, Ho JCW, Ng MMT, Kuok DIT, Shih KC, Tsao SW, Poon LLM, Peiris M, Nicholls JM, Chan MCW (2020). Tropism, replication competence, and innate immune responses of the coronavirus SARS-CoV-2 in human respiratory tract and conjunctiva: an analysis in ex-vivo and in-vitro cultures. Lancet Respir Med..

[18] Wan Y, Shang J, Graham R, Baric RS, Li F (2020). Receptor recognition by the novel coronavirus from Wuhan: an analysis based on decade-long structural studies of SARS coronavirus. J Virol..

[19] Guan WJ, Ni ZY, Hu Y, Liang WH, Ou CQ, He JX, Liu L, Shan H, Lei CL, Hui DSC, du B, Li LJ, Zeng G, Yuen KY, Chen RC, Tang CL, Wang T, Chen PY, Xiang J, Li SY, Wang JL, Liang ZJ, Peng YX, Wei L, Liu Y, Hu YH, Peng P, Wang JM, Liu JY, Chen Z, Li G, Zheng ZJ, Qiu SQ, Luo J, Ye CJ, Zhu SY, Zhong NS, China Medical Treatment Expert Group for Covid-19 (2020). Clinical characteristics of coronavirus disease 2019 in China. N Engl J Med..

[20] Pung R, Chiew CJ, Young BE, Chin S, Chen MI, Clapham HE (2020). Investigation of three clusters of COVID-19 in Singapore: implications for surveillance and response measures. Lancet..

[21] Guan WJ, Liang WH, Zhao Y, Liang HR, Chen ZS, Li YM, Liu XQ, Chen RC, Tang CL, Wang T, Ou CQ, Li L, Chen PY, Sang L, Wang W, Li JF, Li CC, Ou LM, Cheng B, Xiong S, Ni ZY, Xiang J, Hu Y, Liu L, Shan H, Lei CL, Peng YX, Wei L, Liu Y, Hu YH, Peng P, Wang JM, Liu JY, Chen Z, Li G, Zheng ZJ, Qiu SQ, Luo J, Ye CJ, Zhu SY, Cheng LL, Ye F, Li SY, Zheng JP, Zhang NF, Zhong NS, He JX, China Medical Treatment Expert Group for COVID-19 (2020). Comorbidity and its impact on 1590 patients with COVID-19 in China: a nationwide analysis. Eur Respir J..

[22] Catanzaro M, Fagiani F, Racchi M, Corsini E, Govoni S, Lanni C (2020). Immune response in COVID-19: addressing a pharmacological challenge by targeting pathways triggered by SARS-CoV-2. Signal Transduct Target Ther..

[23] Wong CK, Lam CW, Wu AK, Ip WK, Lee NL, Chan IH (2004). Plasma inflammatory cytokines and chemokines in severe acute respiratory syndrome. Clin Exp Immunol..

[24] Rao VUS, Arakeri G, Subash A, Rao J, Jadhav S, Suhail Sayeed M, Rao G, Brennan PA (2020). COVID-19: Loss of bridging between innate and adaptive immunity?. Med Hypotheses..

[25] Mehta P, McAuley DF, Brown M, Sanchez E, Tattersall RS, Manson JJ (2020). COVID-19: consider cytokine storm syndromes and immunosuppression. Lancet..

[26] Chen G, Wu D, Guo W, Cao Y, Huang D, Wang H, Wang T, Zhang X, Chen H, Yu H, Zhang X, Zhang M, Wu S, Song J, Chen T, Han M, Li S, Luo X, Zhao J, Ning Q (2020). Clinical and immunological features of severe and moderate coronavirus disease 2019. J Clin Invest..

[27] Rodriguez-Morales AJ, Cardona-Ospina JA, Gutiérrez-Ocampo E, Villamizar-Peña R, Holguin-Rivera Y, Escalera-Antezana JP, Alvarado-Arnez LE, Bonilla-Aldana DK, Franco-Paredes C, Henao-Martinez AF, Paniz-Mondolfi A, Lagos-Grisales GJ, Ramírez-Vallejo E, Suárez JA, Zambrano LI, Villamil-Gómez WE, Balbin-Ramon GJ, Rabaan AA, Harapan H, Dhama K, Nishiura H, Kataoka H, Ahmad T, Sah R (2020). Clinical, laboratory and imaging features of COVID-19: a systematic review and meta-analysis. Travel Med Infect Dis..

[28] Yang AP, Liu JP, Tao WQ, Li HM (2020). The diagnostic and predictive role of NLR, d-NLR and PLR in COVID-19 patients. Int Immunopharmacol..

[29] Huang C, Wang Y, Li X, Ren L, Zhao J, Hu Y, Zhang L, Fan G, Xu J, Gu X, Cheng Z, Yu T, Xia J, Wei Y, Wu W, Xie X, Yin W, Li H, Liu M, Xiao Y, Gao H, Guo L, Xie J, Wang G, Jiang R, Gao Z, Jin Q, Wang J, Cao B (2020). Clinical features of patients infected with 2019 novel coronavirus in Wuhan , China. Lancet.

[30] Arachchillage DRJ, Laffan M (2019). Pathogenesis and management of thrombotic disease in myeloproliferative neoplasms. Semin Thromb Hemost..

[31] Okamoto K, Tamura T, Sawatsubashi Y (2016). Sepsis and disseminated intravascular coagulation. J Intensive Care..

[32] Levi M, van der Poll T (2010). Inflammation and coagulation. Crit Care Med..

[33] Levi M, van der Poll T, ten Cate H, van Deventer SJ (1997). The cytokine-mediated imbalance between coagulant and anticoagulant mechanisms in sepsis and endotoxaemia. Eur J Clin Invest..

[34] Levi M, van der Poll T (2017). Coagulation and sepsis. Thromb Res..

[35] Leach JL, Fortuna RB, Jones BV, Gaskill-Shipley MF (2006). Imaging of cerebral venous thrombosis: current techniques, spectrum of findings, and diagnostic pitfalls. Radiographics..

[36] Klok FA, Kruip MJHA, van der Meer NJM, Arbous MS, Gommers DAMPJ, Kant KM, Kaptein FHJ, van Paassen J, Stals MAM, Huisman MV, Endeman H (2020). Incidence of thrombotic complications in critically ill ICU patients with COVID-19. Thromb Res..

[37] Abou-Ismail MY, Diamond A, Kapoor S, Arafah Y, Nayak L (2020). The hypercoagulable state in COVID-19: incidence, pathophysiology, and management. Thromb Res..

[38] Zhang Y, Xiao M, Zhang S, Xia P, Cao W, Jiang W, Chen H, Ding X, Zhao H, Zhang H, Wang C, Zhao J, Sun X, Tian R, Wu W, Wu D, Ma J, Chen Y, Zhang D, Xie J, Yan X, Zhou X, Liu Z, Wang J, du B, Qin Y, Gao P, Qin X, Xu Y, Zhang W, Li T, Zhang F, Zhao Y, Li Y, Zhang S (2020). Coagulopathy and antiphospholipid antibodies in patients with COVID-19. N Engl J Med..

[39] Lippi G, Favaloro EJ (2020). D-dimer is associated with severity of coronavirus disease 2019: a pooled analysis. Thromb Haemost..

[40] Tal S, Spectre G, Kornowski R, Perl L (2020). Venous thromboembolism complicated with COVID-19: what do we know so far?. Acta Haematol..

[41] Poterucha TJ, Libby P, Goldhaber SZ (2017). More than an anticoagulant: do heparins have direct anti-inflammatory effects?. Thromb Haemost..

[42] Barrett CD, Moore HB, Moore EE, McIntyre RC, Moore PK, Burke J (2020). Fibrinolytic therapy for refractory COVID-19 acute respiratory distress syndrome: scientific rationale and review. Res Pract Thromb Haemost..

